# Prevalence of obsessive compulsive symptoms among patients with schizophrenia

**DOI:** 10.4103/0972-6748.62263

**Published:** 2009

**Authors:** Smita Hemrom, Pushpa, Divya Prasad, Masroor Jahan, Amool R. Singh, D. K. Kenswar

**Affiliations:** Department of Clinical Psychology, RINPAS, Kanke, Ranchi, Jharkhand, India; 1 Department of Psychiatric Social Work, RINPAS, Kanke, Ranchi, Jharkhand, India

**Keywords:** Compulsion, Obsession, Obsessive compulsive symptoms, Schizophrenia

## Abstract

**Background:**

Obsessive compulsive symptoms in schizophrenia are well recognized but are a less-researched entity. These symptoms have important implications for management and prognosis.

**Aim:**

To find out the prevalence of obsessive compulsive symptoms among patients with schizophrenia.

**Materials and Methods:**

A total of 90 hospitalized patients with schizophrenia diagnosed according to DCR of ICD-10 criteria were selected for the study. Padua inventory and Yale-Brown Obsessive Compulsive Scale were applied to find out the prevalence and nature of obsessive compulsive symptoms.

**Results:**

It was found that 10% of schizophrenic patients had obsessive compulsive symptoms.

**Conclusion:**

Obsessive compulsive symptoms are prevalent in patients with schizophrenia. The presence of comorbidity should be explored for adequate management.

Obsessive compulsive phenomena have been described for more than 100 years in various forms as a part of schizophrenia. The obsessive compulsive symptoms may take the form of contamination; and sexual, religious, aggressive or somatic themes, with or without accompanying compulsions such as cleaning, checking, hoarding, repeating and arranging.

Westphal in 1878 hypothesized that obsessive compulsive syndrome was a variant or a prodrome of schizophrenia. Bleuler thought that some patients suffering from chronic obsessive symptoms were in fact schizophrenic. Strengel (1945) hypothesized a possible interaction between neurotic obsessive compulsive manifestations and psychotic reactions during the course of illness as a part of adaptive defense mechanism. In early studies, Jaherresis (1926) and Rosen (1957) examined clinical records of a large number of schizophrenia cases and found incidence rates of comorbid obsessive compulsive symptoms to be 1.1% and 3.5%, respectively, and concluded that the patients with obsessive compulsive schizophrenia tended to have a comparatively benign clinical course and better outcome. Berman *et al*. (1998) found that a significant number of patients with schizophrenia (up to 50%) have obsessive compulsive-like symptoms coexisting with psychosis, and these symptoms can be easily overlooked by clinicians. Poyurovsky *et al*. (2001) identified 23% prevalence rate in chronic schizophrenia, and Rabe-Jablonska (2001) found 13% prevalence in chronic adolescent schizophrenics. Using Social Behavior Schedule, Poyurovsky *et al*. (2001) reported that those patients with obsession had significantly impaired social functioning. No significant difference was found in a variety of clinical variables, including positive and negative symptoms, abnormal involuntary movements and akathesia, between the obsessive and non-obsessive schizophrenic patients. Turkcan *et al*. (2007) have reported that 16% of schizophrenic patients had obsessive compulsive symptoms. Symptoms were aggression, contamination and sexual obsessions, as well as cleaning compulsions and repetitive rituals.

There has also been an increase in the number of studies addressing the impact that obsession and compulsion have on the symptoms and functioning of those with schizophrenia. Patients displaying comorbid obsessive compulsive disorder and schizophrenia have been shown to have significantly higher positive and emotional discomfort symptoms, poorer executive brain function (Lysaker *et al*., 2000) and reduced capacity for global, social and economic functioning (Fenton, 1986; Tibbo *et al*., 2000).

In India, Jaydeokar *et al*. (1997) did a study to determine the prevalence of obsessive compulsive symptoms among chronic schizophrenic patients. The study revealed that 26.7% of chronic schizophrenic patients had significant obsessive compulsive symptoms, with a high prevalence in the age group ‘below 35 years.’ Obsessive compulsive symptoms were more severe in patients with duration of illness more than 5 years. The obsessive compulsive symptoms were more prevalent among paranoid schizophrenics, with the frequent obsessions being those of contamination, sexual and aggressive thoughts and frequent compulsion or need to ask or confuse. Ganesan *et al*. (2001) studied the phenomenology, treatment, course and outcome of patients with comorbid obsessive compulsive disorder and psychotic features. Results showed that obsessive doubts, washing and checking compulsions were the most commonly seen obsessive compulsive symptoms. Chakraborty *et al*. (2004) found a markedly high incidence of obsessive compulsive symptoms in schizophrenia; 6 (about 31.6%) out of 19 schizophrenic patients had obsessive compulsive symptoms. However, there was no correlation between the psychopathology and presence of obsessive compulsive symptoms. Rajkumar *et al*. (2008) studied the clinical profile of schizophrenic patients with and without comorbid obsessive compulsive disorder. They found that schizo-obsessive patients were more likely to have paranoid symptoms and first-rank symptoms of schizophrenia. They had higher depression scores, more comorbid personality disorders and somewhat lesser disability. Significant correlations were observed between the Yale-Brown Obsessive Scale scores and schizophrenia symptoms dimension scores.

In view of the importance of obsessive symptoms in management and prognosis of schizophrenia, the present study was conducted to find out the prevalence and nature of obsessive compulsive symptoms among hospitalized schizophrenic patients.

## MATERIALS AND METHODS

### Sample

Sample for the study consisted of 90 hospitalized schizophrenic patients (55 men and 35 women) who were diagnosed according to DCR of ICD-10 criteria. Patients were selected from the inpatient units of RINPAS, Kanke, Ranchi, on the basis of purposive sampling technique. Patients of both sexes were enrolled. Participants were within the age range of 20 to 50 years, and the minimum educational level among them was up to the primary level. The duration of the illness was at least 2 years. Patients were excluded if they had comorbid diagnosis of substance dependence, personality disorders, organic disorders or other psychiatric disorders.

The mean age of the patients was 32.4 years. Majority of the patients were male. Most of the patients were educated up to the secondary level. Majority of the patients were married and unemployed. Majority of the patients had a semi-urban background and followed Hindu religion.

### Tools

#### Socio-demographic and clinical data sheet

To collect information regarding socio-demographic characteristics and other related clinical information about the study subjects, a socio-demographic and clinical data sheet was developed.

#### Scale for the assessment of negative symptoms (Andreasen, 1983)

This scale assesses negative symptoms of schizophrenia. It is divided into 7 negative symptoms, namely, affecting flattening or blunting, alogia, avolition, apathy, anhedonia, asociality and inattention. It is a 6-point rating scale comprising of 24 items.

#### Scale for the assessment of positive symptoms (Andreasen, 1984)

This scale assesses positive symptoms of schizophrenia. It is divided into 5 positive symptoms, namely, hallucination, delusion, bizarre behavior, positive formal thought disorders and inappropriate affect. It is a 6-point rating scale comprising of 35 items.

#### Padua inventory (Sanavio, 1988)

This scale assesses phenomenology of, and degree of distress from, obsessive compulsive symptoms. Each item is rated on a 5-point scale, from 0 (not at all) to 4 (very much). There are 4 factors, namely, impaired control over mental activities, becoming contaminated, checking behaviors, and urges and worries of loss of control of motor behavior.

#### Yale-Brown obsessive compulsive scale (Goodman *et al*., 1989)

This scale rates the severity of obsessive compulsive symptoms. The scale is a clinician-rated 10-item scale. Each item is rated 0 (not significant) to 4 (extreme symptoms). Separate total for severity of obsession and compulsion is calculated. The result can be interpreted as 0-7, subclinical; 8-15, mild; 16-23, moderate; 24-31, severe; and 32-40, extreme severity.

### Procedure

After screening, 90 hospitalized schizophrenic patients were selected. Scale for the Assessment of Negative Symptoms (SANS) and Scale for the Assessment of Positive Symptoms (SAPS) were applied to assess the negative and positive symptoms of schizophrenia. Padua Inventory and Yale-Brown Obsessive Compulsive Scale were administered to find out the phenomenological features and severity of obsessive compulsive symptoms.

## RESULTS AND DISCUSSION

The aim of the study was to find out the prevalence of obsessive-compulsive symptoms among patients with schizophrenia. Nine (10%) patients scored above cut off point on Y-BOCS, which suggests that 10% of schizophrenic patients were having significant symptoms of obsessive compulsive disorder. Findings are consistent with the results of prior studies. Thara and Taj (2008) also found that 10.57% of schizophrenic patients had obsessive compulsive symptoms. Rabe-Jablonska (2001) found obsessive symptoms in 13% of chronic adolescent schizophrenics. However, significantly varied range of prevalence rate is reported in few other studies. Few studies reported low prevalence of comorbid obsessive compulsive symptoms in schizophrenia, viz., 1.1% to 3.5% (Jaherresis, 1926; Rosen, 1957); whereas in a few studies, prevalence rate is reported up to 50% (Berman *et al*., 1998).

Obsessive compulsive symptoms on Y-BOCS included contamination; obsession; and washing, checking and counting compulsions. Padua Inventory includes impaired control over mental activities (factor 1), becoming contaminated (factor 2), checking behaviors (factor 3), and urges and worries of loss of control of motor behavior (factor 4). Comparison was done for schizophrenic patients with and without obsessive compulsive symptoms. Out of 81 patients without significant obsessive compulsive symptoms, 9 patients were selected randomly by lottery method to make a comparable group. U test was done to compare these two groups on Y-BOCS and Padua Inventory. Findings are given in Table 1. Findings suggest that there was significant difference in all symptoms except factor 4, which is related to urges and worries of loss of control of motor behavior. Previous studies have also mentioned that types of obsessions and compulsions experienced by patients with schizophrenia are similar to those found in classical obsessive compulsive disorder, e.g., contamination, obsessions, hand washing, repetitive rituals and counting and checking compulsions (Tibbo *et al*., 2000). Turkcan *et al*. (2007) have also reported contamination and sexual obsessions, cleaning compulsions and repetitive rituals in schizophrenic patients.

**Table 1:** Comparison of schizophrenic patients with and without obsessive compulsive symptoms

Scale	Sub-scale	Schizophrenia with obsessive compulsive symptoms (n = 9)		Schizophrenia without obsessive compulsive symptoms (n = 9)		U-value (df = 16)
		Mean	SD	Mean	SD	
Y-BOCS	Obsession scale	10.55	1.6	.33	1.00	.000**
	Compulsion scale	10.55	1.23	.00	.00	.000**
	Total scale	21.11	2.14	.00	.00	.000**
Padua inventory	Factor 1	13.66	7.46	.22	.66	.000**
	Factor 2	13.77	11.20	.00	.00	13.50**
	Factor 3	5.55	8.14	.00	.00	18.00*
	Factor 4	.00	.00	.22	.66	36.00
	Total	36.77	6.33	.66	2.00	.000**

* P < .05

** P < .01

Clinical characteristics of schizophrenic patients with and without obsessive compulsive symptoms were compared. No significant difference was found in age and age at onset of these two groups, however, significant difference was found in duration of illness [Table 2]. Findings suggest that schizophrenic patients with obsessive compulsive symptoms were having significantly longer duration of illness. Jaydeokar *et al*. (1997) also found that obsessive compulsive symptoms in schizophrenic patients are related to duration of illness. These symptoms were more prominent in patients with more than 5 years of total duration of illness.

**Table 2:** Comparison of clinical characteristics of schizophrenic patients with and without obsessive compulsive symptoms

Variable	Schizophrenia with obsessive compulsive symptoms (n = 9)		Schizophrenia without obsessive compulsive symptoms (n = 9)		U-value (df = 16)
	Mean SD	SD	Mean	SD	
Age	33.22	7.04	36.88	7.84	26.50	
Age at onset of	28.77	7.03	29.55	5.93	37.50	
illness				
Duration of	7.55	4.33	3.77	1.39	16.50*	
illness (in years)				

* P < .05

Correlation between positive and negative symptoms and obsessive compulsive symptoms was calculated using Spearman’s Rho [Table 3]. No significant correlation was found between positive and negative symptoms of schizophrenia, and obsessive compulsive symptoms. Similar findings are reported by Berman *et al*. (1998) and Chakraborty *et al*. (2004). In a few studies, significant correlations between positive and negative symptoms, and obsessive compulsive symptoms have been reported; however, findings are contradictory. Lysaker *et al*. (2000) and Rajkumar *et al*. (2008) reported more positive symptoms, and Nechmand *et al*. (2003) reported more negative symptoms in schizophrenic patients with obsessive compulsive symptoms.

**Table 3:** Correlation between clinical features and OC symptoms

	Padua inventory	Y-BOCS
Scale for the assessment of negative symptoms	.280	.397
Scale for the assessment of positive symptoms	.254	.112

## CONCLUSION

Findings of the present study suggest that 10% of schizophrenic patients had significant obsessive compulsive symptoms. Symptoms were related to both obsession and compulsion. Patients had symptoms of impaired control over mental activities, becoming contaminated and checking behaviors. Obsessive compulsive symptoms are significantly correlated with longer duration of illness.

## References

[CIT1] Andreasen N. C (1983). The scale for the Assessment of Negative Symptoms (SANS).

[CIT2] Andreasen N. C (1984). The scale for the Assessment of Positive Symptoms (SAPS).

[CIT3] Berman I, Merson A, Viegner B (1998). Obsessive and compulsions as a distinct cluster of symptoms in schizophrenia: A neuropsychological study. Journal of Nervous and Mental Diseases.

[CIT4] Chakraborty R, Chatterjee A, Bakhala A, Singh A. R, Chakraborty P. K (2004). Obsessive compulsive symptoms in major psychoses. Indian Journal of Clinical Psychology.

[CIT5] Fenton W. S (1986). The prognostic significance of obsessivecompulsive symptoms in schizophrenia. American Journal of Psychiatry.

[CIT6] Ganesan V, Ramesh Kumar T. C, Khanna S (2001). Obsessive-Compulsive Disorder and Psychosis. Canadian Journal of Psychiatry.

[CIT7] Goodman W. K, Price L. H, Rasmussen S. A (1989). The Yale-Brown Obsessive Compulsive Scale. II. Validity. Archives of General Psychiatry.

[CIT8] Jharrreiss W (1926). Obsessions during schizophrenia. Archives of Psychiatry.

[CIT9] Jaydeokar S, Gore Y, Diwan P, Deshpande P, Desai N (1997). Obsessive-compulsive symptoms in chronic schizophrenia: A new idea or an old belief?. Indian Journal of Psychiatry.

[CIT10] Lysaker P. H, Marks K. A, Picone J. B, Rollins A. L, Fastenau P. S, Bond G. R (2000). Obsessive and compulsive symptoms in Schizophrenia: Clinical and neurocognitive correlates. Journal of Nervous and Mental Diseases.

[CIT11] Nechmad A, Ratzoni G, Poyurovsky M, Meged S (2003). Obsessive-compulsive disorders in adolescent schizophrenia patients. American Journal of Psychiatry.

[CIT12] Poyurovsky M, Hramenkov S, Isakov V (2001)). Obsessive compulsive disorder in hospitalized patients with chronic schizophrenia. Psychiatry Research.

[CIT13] Rabe-Jablonska J (2001). Obsessive compulsive disorders in adolescents with diagnosed schizophrenia. Psychiatria Polska.

[CIT14] Rajkumar R. P, Reddy Y. C. J, Kandavel T (2008). Clinical profile of “schizo-obsessive” disorder: A comparative study. Comprehensive Psychiatry.

[CIT15] Rosen I (1957). The clinical significance of obsession in schizophrenia. Journal of Mental Sciences.

[CIT16] Sanavio E (1988). Obsession and compulsion: The Padua Inventory. Behaviour Research and Therapy.

[CIT17] Strengel E (1945). A study of some clinical aspects of the relationship between obsessional neurosis and psychotic reaction types. Journal of Mental Science.

[CIT18] Thara R, Taj M (2008). Obsessive- Compulsive symptoms in schizophrenia. Eastern Journal of Psychiatry.

[CIT19] Tibbo P, Kroetch M, Chue P, Warneke L (2000). Obsessive-compulsive disorder in Schizophrenia. Journal of Psychiatry Research.

[CIT20] Turkcan A, Yanbay H, Satmis N, Ceylan M. E (2007). Obsessive- compulsive symptoms in inpatients with schizophrenia: A preliminary study. Bulletin of Clinical Psychopharmacology.

